# Enabling patients with advanced chronic obstructive pulmonary disease to identify and express their support needs to health care professionals: A qualitative study to develop a tool

**DOI:** 10.1177/0269216319833559

**Published:** 2019-03-05

**Authors:** A Carole Gardener, Gail Ewing, Morag Farquhar

**Affiliations:** 1 Primary Care Unit, Department of Public Health and Primary Care, University of Cambridge, Cambridge, UK; 2 Centre for Family Research, University of Cambridge, Cambridge, UK; 3 School of Health Sciences, University of East Anglia, Norwich, UK

**Keywords:** Chronic obstructive pulmonary disease, needs assessment, patient-centred care, qualitative evidence-based practice, communication

## Abstract

**Background:**

Patients with advanced chronic obstructive pulmonary disease have difficulty reporting their holistic support needs to health care professionals, undermining delivery of person-centred care. We lack tools that directly support patients with this.

**Aim:**

To develop an evidence-based, designed-for-purpose, tool to enable patients to directly identify and express support needs to health care professionals.

**Design:**

Two-stage qualitative study. Stage 1: domains of support need were identified through a systematic review, analysis of an established qualitative dataset and patient/carer focus groups. Stage 2: draft tool developed using the identified domains of need and then refined through feedback from patients, carers and health care professionals, ensuring acceptability and suitability.

**Setting/participants:**

Stage 1 patients/carers recruited via four primary care practices and two patient support groups (East of England). Stage 2 health care professionals recruited via the Clinical Research Network and local community trust and patients/carers through two further practices and two additional support groups (East of England). In total, 57 patients, carers and health care professionals participated.

**Results:**

A comprehensive set of evidence-based support domains (for example: overcoming boredom or loneliness, knowing what to expect in the future) was identified and formulated into questions. The resulting tool asks patients to consider whether they need more support in 15 broad areas. Patients, carers and clinical stakeholders broadly endorsed the tool’s content and wording.

**Conclusion:**

The Support Needs Approach for Patients (SNAP) tool is a concise evidence-based tool designed to help patients with advanced chronic obstructive pulmonary disease identify and express their support needs to enable delivery of person-centred care.

**What is already known about this topic?**

- Patients with advanced chronic obstructive pulmonary disease (COPD) have unmet support needs, arising from patient difficulties in identifying and expressing their needs compounded by health care professional and organisational barriers.
- Policy makers recommend a holistic, person-centred approach to improving care for patients with long-term conditions, emphasising that patient-identified need should inform delivery of care.
- We currently lack appropriate tools to help patients directly to identify and express their support needs to health care professionals.

**What this paper adds?**

- Development of an evidence-based designed-for-purpose tool to help patients directly identify and express their support needs.
- Fifteen evidence-based domains of support need including, for example, overcoming boredom or loneliness, knowing what to expect in the future and looking after any other physical health problems.

**Implications for practice, theory or policy**

- The developed tool is designed to support delivery of a holistic, person-centred approach to identifying and responding to need.

## Introduction

People with advanced long-term conditions, such as chronic obstructive pulmonary disease (COPD), experience disabling physical symptoms frequently combined with high psychological and social distress.^1–4^ The need to deliver holistic, supportive, needs-led, person-centred care to patients is internationally recognised.^5–8^ This requires involving patients in identifying and addressing their support needs, i.e. those aspects of managing life with which they may need support (e.g. to manage symptoms or address financial concerns).^6,8,9^ However, there remain high levels of unmet support need in patients with advanced COPD, and provision of person-centred care is highly variable.^10–13^

Against this international backdrop, UK guidelines for patients with long-term conditions and palliative and end-of-life care^14–17^ advocate using tools to enable patient involvement in identifying their needs and preferences. Examples of recommended tools are: PEPSI COLA Aide Memoire,^18^ Holistic Common Assessment (HCA) tool,^16^ Well-Being Star,^19^ Distress Thermometer and Problem List,^20^ Kirklees Health Needs Assessment tool^21^ and Sheffield Profile for Assessment and Referral to Care (SPARC).^22^ However, these tools primarily focus on measuring disease burden, patient functionality or patient concerns – these can be valuable indicators of need but do not directly identify areas where patients need more support to manage life with their condition. Some focus only on a narrow range of support domains or prescribed responses to need (e.g. information), limiting consideration of wider support needs (e.g. SPARC). In addition, the length of some tools can undermine their utility for patients with advanced COPD, for example, the Kirklees HNA. Furthermore, tools such as the HCA and PEPSI COLA Aide Memoire are practitioner-led, contrasting with patient-completed tools that actively support a person-centred approach through ensuring that areas of support need identified and discussed are those prioritised by patients. There is also growing interest in using patient-reported outcome measures to facilitate discussion of patient needs,^23^ but these suffer from similar limitations to the above, plus their weighting towards medical symptoms means they are potentially more useful for health care professionals in relation to treatment decisions or measuring outcomes than for person-centred comprehensive identification of need. Thus, this study aimed to develop an evidence-based, designed-for-purpose, tool to enable patients directly identify and express their support needs to health care professionals.

## Methods

**Figure 1.**
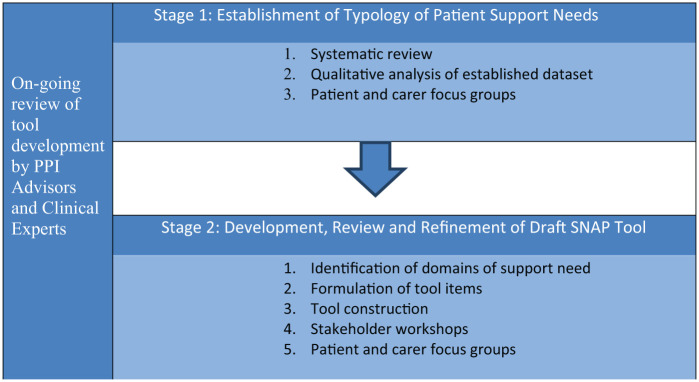
Two stages of Support Needs Approach for Patients (SNAP) tool development.

To develop the tool, an in-depth understanding of patient support needs was required from the perspectives of patients themselves, illuminating their experiences, therefore a qualitative approach was adopted. The study comprised two stages, as illustrated in Figure 1: (1) establishment of a comprehensive evidence-based typology of patient support needs in advanced COPD and (2) development and refinement of the tool. In addition, public and patient involvement (PPI) advisors and clinical experts iteratively reviewed the developing tool to ensure acceptability and suitability for patients and clinical practice.

### Ethics

Ethical approval was obtained from the following:

1. East of England–Cambridge South Research Ethics Committee: Ref.16/EE/0064 (29 March 2016: for Stage 1, and for the Stage 2 stakeholder workshops).
2. East of England–Essex Research Ethics Committee: Ref.17/EE/0192 (17 May 2017: for the Stage 2 patient and carer focus groups).

Written informed consent was obtained from all patients, carers and participating health care professionals.

### Stage 1 – establishment of an evidence-based typology of patient support needs

Three sources of evidence were used to establish the typology of patient support needs in advanced COPD: (1) a systematic review of the literature, (2) further analysis of purposively sampled data from an established dataset and (3) new patient and carer focus groups.

1. Systematic review.

A systematic search and narrative review of published literature identified known support needs in COPD and is reported in detail elsewhere.^24^ In brief, 31 papers were reviewed and 13 domains (broad areas) of support need were identified: (1) understanding COPD, (2) managing symptoms and medication, (3) healthy lifestyle, (4) managing feelings and worries, (5) living positively with COPD, (6) thinking about the future, (7) anxiety and depression, (8) practical support, (9) finance work and housing, (10) families and close relationships, (11) social and recreational life, (12) independence and (13) navigating services.^24^

2. Further analysis of purposively sampled data from an established dataset.

#### Dataset characteristics

Transcripts of baseline interviews with 20 patients with advanced COPD, or patient-carer dyads, conducted within the Living with Breathlessness Study between January and November 2013 (LwB: Improving Care and Support in Advanced COPD),^25^ were purposively sampled for further analysis (with the approval of East of England–Cambridge South Research Ethics Committee: Re.16/EE/0064). Characteristics of the parent study and parameters to generate the purposive sample are outlined in Box 1.

**Box 1.** Parent study characteristics and inclusion and exclusion criteria, and purposive sampling parameters for development of a typology of support needs.

Living with Breathlessness Study – Longitudinal Interview Study (LIS) characteristics	LIS inclusion criteria	LIS exclusion criteria	Purposive sampling parameters to identify LIS transcripts for typology development
Population-based longitudinal mixed method cohort study Recruitment via East of England primary care practices Recruited: 235 patient and 115 informal carers Audio-recorded mixed methods interviews (in participants’ location of choice) Quantitative data: demographics, comorbidities and service use and disease-specific health-related quality of life and psychological health using validated questionnaires Qualitative data: living with advanced chronic obstructive pulmonary disease (COPD), self-identified need, views on formal and informal care and thoughts on future care	Patients with COPD meeting two or more of the following: FEV1 < 30% 2+ exacerbations requiring prednisolone and antibiotics in the previous year Long-term oxygen therapy Cor pulmonale MRC dyspnoea scale 4+ Admission for COPD in previous year	Patients with any of the following: Serious mental health problem Serious learning difficulty Active cancer Active alcoholism	Sex Patient has/had no informal carer Location of patient (rural/urban) High/low levels of psychological morbidity (Hospital and Anxiety Depression Scale)^26^ High/low levels of disease impact (COPD Assessment Test)^27^ Patient has/had no patient-identified key health care professional (self-report)

#### Data analysis

Thematic analysis using the Framework Approach,^28^ facilitated by NVivo,^29^ was used to identify key aspects of support identified by patients. The 13 support domains identified by the systematic review provided the basis for the coding frame.

To enable identification of additional support needs emerging from the data, but not found in the review, two approaches were taken: (1) addition of an ‘other’ category to the coding frame and (2) inclusion of mechanisms for expressing support needs following Ewing and Grande’s^30^ framework of support needs that were met (‘met needs’), supportive input that was perceived as helpful (‘helpful input’) and shortfalls in provision where needs had not been met (‘unmet needs’).

Data were extracted into the coding frame and analysed by A.C.G. and M.F. The findings provided the draft typology of patient support needs, which was applied to a further random sample (*n* = 20) of the remaining baseline interviews from the parent study (*n* = 215), to establish comprehensiveness.

3. Patient and carer focus groups.

Focus groups involving both patients and carers were conducted to review the draft typology of support needs in advanced COPD developed from the systematic review and the results of the analysis of purposively sampled data from the Living with Breathlessness Study.

#### Recruitment of focus group participants

Four primary care practices in the East of England (two rural and two urban, recruited via the Clinical Research Network (CRN)) identified patients against the same inclusion and exclusion criteria outlined in Box 1. Eligible patients were posted recruitment packs by practices (invitation letter, participant information sheet, reply form and prepaid envelope for its return), which also invited patients to bring along a family member/friend who supported them. Recruitment was further facilitated by two British Lung Foundation Breathe Easy support groups using identical packs. Those interested in participating returned completed reply forms directly to the research team, giving their contact details; they were then telephoned to answer any questions and make arrangements for the focus groups.

#### Data collection

Three focus groups were conducted (June and July 2016), with four to six participants in each. Groups took place in local hotel meeting rooms chosen for ease of access and comfort, lasted approximately one hour, and were audio-recorded with permission. Each group was facilitated by A.C.G., M.F. and G.E. Participants were provided with lunch and completed a brief demographic questionnaire. G.E. was available to support any distressed participants during discussions, however, none required support. Participant information sheets provided contact details for post-group support (local Community Respiratory Team lead and highly experienced senior nurse); again, none was required.

Groups started by asking participants to look at the draft typology of support needs and identify individual domains particularly important to them and explain why. They then discussed important aspects of support received, forms of support they would like but not had access to and support at critical times (received or not). In the final activity, they revisited the draft typology of support need, discussed the relevance of each need and considered any support needs not covered.

#### Data analysis

Audio recordings were fully transcribed, checked for accuracy and anonymised. Transcripts were read for familiarisation and a (conventional) content analysis^31,32^ conducted by A.C.G., M.F. and G.E. to produce a final typology of patient support needs in advanced COPD.

### Stage 2 – development, review and refinement of the patient support needs tool

The final typology of support needs was formulated into evidence-based items for the draft patient support needs tool. Layout of the draft tool was modelled on the well-established and tested Carer Support Needs Assessment Tool (CSNAT).^30,33–35^

The draft tool was reviewed and refined in an iterative process involving (1) stakeholder workshops and (2) patient and carer focus groups, as outlined in Box 2. In brief, patients and carers were recruited to stakeholder workshops from two further Breathe Easy support groups using processes outlined in Stage 1, with workshops held in their usual meeting place (community centres). Health care professionals were recruited to stakeholder workshops via the CRN (two practices) and local community trust (one community respiratory team), with workshops conducted on site. Patients and carers were recruited to Stage 2 focus groups via two further primary care practices in using processes outlined in Stage 1, with focus groups held in local hotels. All Stage 2 stakeholder workshops and focus groups were facilitated by A.C.G. and M.F. and took place between June 2016 and July 2017. Data were recorded, processed and analysed as for Stage 1.

**Box 2.** Methods for Stage 2 review and refinement of the draft support needs tool.

1. Stakeholder workshops			
	Patients and carers	Health care professionals	
Recruitment Recruitment packs sent to:	2× British Lung Foundation Breathe Easy support groups	2× primary care practices	1× Community Respiratory Team
Workshops No. of workshops: No. of participants:	2× patient and carer 10 patients and 5 carers	2× primary care 7 GPs, 2 practice nurses and 1 health care assistant	1× community 1 clinical team manager, 3 respiratory specialist nurses and 1 respiratory physiotherapist
Data collection	Presentation of draft tool to workshop participants Discussion of acceptability and suitability of the following: layout item wording response categories structure introductory instructions tool name suitability for clinical practice		
Data analysis	Audio recordings fully transcribed, checked for accuracy and anonymised. Transcripts read for familiarisation. Content analysis to assess the suitability and acceptability of layout, item wording and response categories. Potential areas for refinement identified.		
2. Patient and carer focus groups			
*Recruitment* Recruitment packs sent to:	44 patients (who met the inclusion/exclusion criteria outline in Box 1) via 2× primary care practices		
Focus groups No. of groups: No. of participants:	2× patient and carer focus groups 12 patients and carers		
Data collection	Participants were asked to comment on the layout, content and utility of the draft tool, to explore face validity and initial content validity of the tool.		
Data analysis	Data were processed and analysed as above.		
Tool refinement	Tool refined by study team based on workshop and focus group findings.		
PPI and Clinical Expert Advisory Groups			
Review of developing tool	Multidisciplinary Study Advisory Group and PPI advisors reviewed and refined the tool for suitability and acceptability for patients and clinical practice		

## Results

### Stage 1 – establishment of an evidence-based typology of patient support needs

#### Focus group sample

Ten patients and five carers agreed to take part (15% response rate based on the number of packs distributed to practices and support groups). Six participants were male with age range 65–87 years. Four carers were spouses (three wives, one husband); one was a community supporter.

#### Typology of patient support needs

Stage 1 analysis of the purposively sampled established qualitative dataset and new patient/carer focus groups identified 24 domains for the final typology of patient support needs outlined in Table 1. Two domains, ‘support for carers’ and ‘looking after other health problems’, were not in the systematic review but emerged purely from the purposively sampled established dataset via the ‘other’ category on the coding frame. In contrast, there was no evidence within the established dataset for the support domain relating to ‘work’, which was only found in the systematic review. Table 1 shows where and how evidence for the 24 domains was identified and how some aspects of support (e.g. ‘financial’ and ‘access to aids and adaptations’) contributed to multiple domains.

**Table 1.** The development of broad areas of support need in advanced COPD.

Coding framework (derived from systematic review)	Key aspects of support identified from the qualitative data within the cross-cutting themes		Domains of support need in advanced COPD (*n* = 23)
	Met needs/unmet needs	Helpful input	
Understanding COPD	Better understanding of the nature of COPD	Discussion of prognosis with a doctor Update on latest information about COPD from health care professionals Information sessions at pulmonary rehabilitation Regular ‘open’ discussions with health care professionals	Understanding COPD
Managing symptoms and medication	Support to manage tiredness Understanding how to manage breathlessness Knowing when to contact services Knowing how to use oxygen Understanding how to manage/control breathing Understanding current state of health Having access to oxygen and nebulisers	Being kept up to date with current state of health Someone to come out to patient and advise about breathlessness Having a nebulizer Proactive contact from health care professionals to provide monitoring and reassurance Input from respiratory specialists concerning inhaler use, managing breathlessness and panic attacks	Managing symptoms and medication
Healthy lifestyle	Support to exercise Support to stop smoking Support to overcome the cycle of weight gain due to side effects and reduced capacity to exercise	Pulmonary rehabilitation Physiotherapy Exercise classes Information about exercising safely Self-motivation to keep active/walking Smoking cessation including patches and encouragement Support from family and friends Non-judgmental services (regarding smoking) Dietician	Staying active Stopping smoking Healthy eating
Managing feelings and worries	Overcoming fears Relief from stress Reassurance that someone is there who can help when needed Practical support to deal with sources of stress (finances/difficult situations/practical concerns)	Support from family, friends and carers Positive thinking Information Carer managing situations the patient finds overwhelming	Managing feelings and worries
Living positively with COPD	Dealing with concerns about the nature of COPD Help to overcome the sense that patient is facing COPD on their own Making sense of the experience of COPD	Peer support group (talking to people with COPD to share difficulties and expertise)	Living positively with COPD
Anxiety and depression		Someone to talk to who understands Psychiatrist Therapist Antidepressants Practical support Specialist respiratory nurses Information/strategies on how to control panic attacks	Overcoming anxiety and depression
Finance, work and housing	Help with paperwork Help sorting out bills and benefits Help to improve finances Financial assistance to help pay for heating, clothing and food	Help sorting out finances Welfare benefits Supported bungalow Downstairs bathroom Relocating to ground floor accommodation	Finance Housing Work (no evidence in qualitative data) Legal
	Accessible or sheltered housing Housing adaptations: wooden floors, wet room, downstairs toilet, stairlift Ability to move closer to family and support networks Support to find out about, or access, housing needs	Existing housing adapted to patient needs	
Families and close relationships	Support talking to relatives about COPD Ongoing mutual support (between patient and relatives)		Talking to relatives about COPD Maintaining positive relationships with families and friends
Independence	Resources to facilitate leaving the house, remaining independent and maintaining mobility Adaptations to facilitate getting the wheelchair out of the house Accessing benefits for parking and transportation. Someone to take patient out (shopping, social activities)	Wheelchair that facilitates going on holiday Mobility scooter that is both easy to transport and sturdy Access to disabled parking Accessible services Ramps Bus pass Someone taking the patient shopping Mobility scooter Family accompanying the patient to medical appointments Home visits from health care professionals Public transport Contact with health care professionals via phone Friend who provides lifts in the car Financial support with transport and car purchase Community transportation Assistive devices and adaptations, e.g. wet room/shower room Doing as much for self as possible Equipment (assistive and adaptive devices, e.g. stairlift, mobility scooters) Computers/iPads	Getting out and about Aids and adaptations
Social and recreational life	More social contacts Practical and financial support to access resources Someone else to drive Access to community groups	Clubs Support groups Family and friends who support attendance at social activities Involvement in family activities and interests Support groups Self-focus on maintaining interest and activities Helpline Computers/tablets and so on	Maintaining activities and interests
Thinking about the future	Help thinking through end of life plans	Pulmonary rehabilitation Discussion about what patient would like to happen Discussion of prognosis with doctor Discussion of DNR with family and doctor	Future planning
Practical support	Cooking dinner and making drinks Changing the bed Gardening Heavy lifting DIY and household maintenance Financial support to access cleaner and carers Concern about strain on carer taking on practical support role Additional support during an exacerbation Some help with personal care, e.g. foot care, showering, washing back	Family taking on practical roles (feeding and walking the dog, running errands, gardening, heavy jobs, housework and shopping) Home carers, cleaners and gardeners Ready cooked meals and microwaves Adaptations to the home (wet rooms and shower rooms) Carer support with bathing, dressing, having a shower, walking, during the night, cutting up food and feeding Self-pacing strategies Home care Carer collecting medications	Practical support in the house or garden Support with personal care
Navigating services	Support managing paperwork related to service use Advocacy: someone to help patient remember what was said in an appointment and put forward patient views Information about services Knowing how to access support in an emergency or if health deteriorates	Carer willing to deal with other people and health care professionals so that patient doesn’t have to Being able to call the GP or a nurse to request advice or home visit Pendant alarm and telehealth Proactive follow-up after emergencies Named health care professional contact Family and friends	Accessing and using services Knowing who to contact
Other (not identified in the systematic review): Looking after other health problems	Support to manage contraindications Understanding from specialist health care professionals and professional carers about wider health needs	Health care professional who understands the whole picture Telehealth service Treatment for other conditions Contact with (individual) specialist services Health care professional for comorbidities Good liaison between GP and specialist services Generic services that cater for variety of needs Community matron Comprehensive review of health	Looking after other physical health problems
Other (not identified in the systematic review): Support for carers	More support for carer in undertaking practical tasks in the home Planned carer breaks Support to enable carers to achieve own goals (work, etc.) Support to help carers understand COPD	Respite care Additional support from extended Use of professional carers Carer included in consultations with health care professionals Equipment to support carers manage practical tasks	Support for carers

COPD: chronic obstructive pulmonary disease.

### Stage 2 – development, review and refinement of patient support needs tool

#### Draft tool items

The 24 support domains from the final typology were synthesised into 16 items for inclusion in the draft tool. The first two columns of Table 2 map this synthesis of the support domains from the typology of need (column 1) into the items for the draft tool (column 2). The table shows that most tool items were derived directly from a single support domain, for example, the domain ‘understanding COPD’ was formulated directly into ‘Do you need more support with understanding your illness?’ A few support domains were combined into one item and a few contributed to more than one item. Decisions to include, combine or divide support domains were discussed and agreed within the research team. An additional item was added to enable patients to note any other support needs they had that they felt may not be covered by the 16 items.

**Table 2.** Item mapping for the development of a patient support needs tool for patients with advanced COPD.

Typology of support needs (24 domains of support need)	Draft tool items (16 items taken to workshops) *Do you need more support with …?*	Draft tool items (15 items taken to Stage 2 patient and carer focus groups) *Do you need more support with …?*	Tool items included in the final version of the SNAP tool (*n* = 15) *Do you need more support with …?*
Understanding COPD	**… understanding your illness**	**… understanding your illness**	… understanding your illness
Managing symptoms and medication	**… managing your symptoms (including medication and oxygen)**	**… managing your symptoms (including medication and oxygen)**	… managing your symptoms (including medication and oxygen)
Staying active	**… having a healthier lifestyle (e.g. keeping active or eating well)**	**… having a healthier lifestyle (e.g. keeping active or eating well)**	… having a healthier lifestyle (e.g. keeping active or eating well)
Stopping smoking			
Healthy eating			
Managing feelings and worries	**… dealing with your feelings and worries**	**… dealing with your feelings and worries**	… dealing with your feelings and worries
Living positively with COPD			
Overcoming anxiety and depression			
Looking after other health problems	**… looking after any other physical health problems you may have**	**… looking after any other physical health problems you may have**	… looking after any other physical health problems you may have
Finance	**… financial, legal, work or housing issues**	**… financial, legal, work or housing issues**	… financial, legal, work or housing issues
Housing			
Work (evidenced only in the systematic review)			
Legal			
Accessing and using services	**…** making services work for you (e.g. accessing services or using services)	**… accessing or using services**	**… accessing or using services**
Knowing who to contact	**…** knowing who to contact if you are concerned		
Aids and adaptations	**…** equipment to help you	… equipment to help you	**… aids or equipment to help you**
Getting out and about	**…** getting out and about	… getting out and about	… getting out and about
Maintaining activities and interests	**… overcoming boredom or loneliness**	… overcoming boredom or loneliness	… overcoming boredom or loneliness
Maintaining positive relationships with families and friends	**… family relationships (including talking to your relatives about your illness)**	**… family relationships (including talking to your relative about your illness)**	**… family relationships (including talking to your relative about your illness)**
Talking to relatives about COPD			
Future planning	**… knowing what to expect in the future**	**… knowing what to expect in the future**	… knowing what to expect in the future
Practical help in the home or garden	**… practical help in the home or garden**	**… practical help in the home or garden**	… practical help in the home or garden
Support with personal care	**… your personal care (e.g. dressing, washing)**	… your personal care (e.g. dressing, washing)	… your personal care (e.g. dressing, washing)
Support for carers	Does your carer (family member or friend who helps you) need more support?	Does your carer (family member or friend who helps you) need more support?	**Does a family member or friend who helps you need more support?**

COPD: chronic obstructive pulmonary disease.

Bold entries in cells represent the establishment of the final version of the SNAP tool items.

#### Draft tool construction

The 16 draft items were then incorporated into a grid layout with three response categories (no/a little more/quite a bit more) to encourage *any* expression of need. The draft tool was titled with the question ‘How are you?’ and brief instructions on how to complete it were added. The overall format was designed to enable ease of completion by patients.

#### Tool review and refinement

1. *Stakeholder workshops.*

Stakeholder workshops with 30 patients, carers and health care professionals broadly endorsed the draft tool’s overall structure. They liked the inviting title, simple layout, concise instructions and straightforward language used: they considered it easy to understand and complete.

I mean, you don’t want ‘questionnaire’ or ‘survey’ … or ‘needs assessment’ … which is terrible [in a title]. But having that [How are you?] …, it’s friendly, it’s fine. (S1, HCP-W1)

Stakeholders identified two areas for improvement: (1) the length of the 16-item draft tool and (2) lack of patient-friendly design (some felt it looked too much like a questionnaire, which could be off-putting). Two items, ‘accessing services’ and ‘knowing who to contact’, were therefore combined, reducing the number of items to 15 (see column 3, Table 2). In addition, an NHS trust media studio was commissioned to produce a patient-friendly version of the tool, in booklet form, incorporating an exemplar cover with space for additional patient and provider information and use of colour.

2. *Patient and carer focus groups.*

Twenty-seven patients were identified by participating primary practices as eligible and invited to take part in Stage 2 focus groups; eight patients and four carers agreed to take part. Seven were female with age range 51–90 years. Carers include two spouses, a daughter and a friend.

Stage 2 focus groups participants reviewed the revised tool and responded positively to the content and new layout:

They’re all relevant questions. (S2, FG1)

It’s all quite clear and straightforward. (S5, FG1)

However, two changes to item wording were recommended. On the item ‘Do you need more support with equipment?’, participants suggested including the word ‘aids’ as this was more commonly used. Regarding the item ‘Does your carer (family member or friend who helps you) need more support?’, for some ‘carer’ meant paid professional care – using the phrase ‘family members or friends who help you’ was felt more appropriate. These two changes were incorporated, and this final version adopted (see column 4, Table 2).

#### PPI advisors and clinical experts

Throughout the review and refinement process, PPI advisors and additional clinical experts drawn from the study’s advisory group reviewed the developing tool for suitability and acceptability for patients and clinical practice. Both groups endorsed the final version of the tool.

## Discussion

### Results of the study

This article describes the two-stage development of a tool to enable patients with advanced COPD to identify and express their support needs. Ultimately the tool will underpin a person-centred approach to care: the Support Needs Approach for Patients (SNAP). The tool is therefore referred to herein as ‘the SNAP tool’. To our knowledge, the SNAP tool is the first concise, evidence-based, designed-for-purpose, tool to help patients directly identify and express their support needs to health care professionals. Furthermore, the evidence-based typology, which informed the tool’s items, outlines for the first time the comprehensive domains of support need for patients with advanced COPD.

### Strengths and weaknesses

A key strength of this study was the use of multiple sources of data enabling us to build on our existing understanding of patient support needs in COPD^24^ by comprehensively identifying the range of support needs in patients in the advanced stage of the disease. It is noteworthy that analysis of qualitative data from an existing dataset from patients with advanced COPD identified two additional areas of support need not found in the systematic review: ‘looking after other health problems’ and ‘support for informal carers’. Similarly, ‘work’ was identified in the review but not the qualitative data. The qualitative data were generated by patients with advanced disease, whereas the review considered patients’ needs at any disease stage, due to the limited number of relevant papers on advanced disease. High levels of comorbidity are experienced by patients with advanced COPD,^36^ and there is an increasing role for informal carers (and associated carer burden and need) as disease progresses;^37,38^ furthermore, few patients with advanced disease work.^39^ Use of multiple data sources therefore ensured a comprehensive evidence base for SNAP tool items.

A further strength was the active role of PPI advisors who reviewed and commented on the developing SNAP tool in their study advisory role. Iterative integration of feedback from patients and carers (both as PPI and as participants) and clinical stakeholders gave confidence on SNAP tool relevance and suitability for patients living with advanced COPD.

A potential limitation of the tool development process was that qualitative data came only from patients from the East of England. However, it is reassuring that the systematic review included national and international data.^24^ Furthermore, the review’s necessary inclusion of studies relating to all stages of COPD is also a strength, as patients and health care professionals have suggested the tool’s utility throughout the disease trajectory. Health care professionals have further suggested the tool’s utility in other disease groups given the broad nature of the domains of support need included (probably reflecting the multimorbid nature of life with advanced COPD) and that the tool does not include language specific to lung disease.

### What this study adds

The evidence-based typology outlines for the first time the comprehensive domains of support need for patients with advanced COPD. It is noteworthy that we found no evidence of patient need for support in relation to spirituality, in either the systematic review^24^ or established dataset analysis, which is known to be important in end-of-life care in cancer.^40^ This may reflect others’ findings that patients with long-term non-malignant conditions perceive of themselves as ‘living with’, rather than ‘dying from’, their conditions,^41^ with implications for the application of ‘one size fits all’ guidelines for palliative and end-of-life care.

The tool’s concise format is designed for compatibility with busy clinical settings, contrasting with existing tools that are too lengthy or are brief but lack comprehensiveness. Furthermore, as a tool that directly identifies patient support needs, the SNAP tool addresses concerns about the use of tools that are indicators of need (such as patient-reported outcome measures) as prompts for discussions about support needs. Indirect indicators of need assume that patients consider there is legitimacy in discussing their support needs with health care professionals, that they understand the holistic nature of supportive care and have the opportunity and confidence to contribute to discussions on what they require to manage life with their illness. Evidence suggests this is not always the case^42,43^ and therefore there is a need for designed-for-purpose tools, such as the SNAP tool. The SNAP tool has therefore been specifically developed to facilitate delivery of a holistic, person-centred approach for the identification of patient support needs: the Support Needs Approach for Patients (SNAP).

Future work will validate the SNAP tool (with patients with advanced COPD) and develop the SNAP intervention underpinned by the tool. It will then explore the feasibility and effectiveness of SNAP in enabling person-centred care in clinical practice by identifying and addressing patients unmet support needs in a range of settings.

The SNAP tool is protected by copyright. It can be viewed in full by requesting an inspection copy via the SNAP website, where the process of obtaining a licence to use the tool (in clinical practice or in research) can also be accessed: theSNAP.org.uk.

## Conclusion

This study outlines the development of an evidence-based, designed-for-purpose, tool to help patients with advanced COPD identify and express their support needs to health care professionals. The developed SNAP tool is distinct from existing patient needs assessment tools in that it is (1) comprehensive, yet concise, and (2) helps patients *directly* identify and express areas where they may require more support to manage life with advanced COPD. The SNAP tool now requires validating before it can be used in clinical practice to enable delivery of person-centred care through the SNAP intervention. For further information about SNAP, please see the SNAP website (theSNAP.org.uk/), contact SNAP.team@uea.ac.uk or follow SNAP on Twitter: @SNAPstudyteam.
